# Open surgical management of pediatric hepatic hydatid cysts: a retrospective cohort study

**DOI:** 10.1097/MS9.0000000000005521

**Published:** 2026-08-25

**Authors:** Amin Sabourifard, Ahmad Mohammadipoor, Mostafa Abrishami, Sahar Soleimanian Moghaddam, Malihe Aghasizadeh, Khashayar Atqiaee

**Affiliations:** a Department of Surgery, Faculty of Medicine, Mashhad University of Medical Sciences, Mashhad, Iran; b Department of Pediatric Surgery, Faculty of Medicine, Mashhad University of Medical Sciences, Mashhad, Iran; c Student Research Committee, Faculty of Medicine, Mashhad University of Science, Mashhad, Iran; d Department of Vascular and Endovascular Surgery, Vascular and Endovascular Surgery Research Center, Mashhad University of Medical Sciences, Mashhad, Iran

**Keywords:** echinococcosis, hepatic, hydatid cyst, treatment outcome

## Abstract

**Background:**

Cystic echinococcosis (CE), caused by *Echinococcus granulosus*, remains a major public health concern in endemic regions such as Iran. The liver is the most affected organ in children, and surgical intervention is often required due to the risk of complications and recurrence.

**Objective:**

To evaluate the outcomes of open surgical treatment for hepatic hydatid cysts in pediatric patients managed at two tertiary pediatric hospitals.

**Methods:**

A retrospective cohort analysis was conducted on 102 children who underwent open surgery between 2017 and 2024. Data were retrospectively collected from medical records and included demographic characteristics, cyst features, surgical techniques, liver function tests, vital signs, and postoperative outcomes. Surgical procedures included intracystic drainage, partial cystectomy, and simple cystectomy. Statistical analysis was performed using SPSS version 24.

**Results:**

Among the 102 pediatric patients, 62.7% were male, with a mean age of 9.08 years. The right hepatic lobe was affected in 66.7% of cases. Surgical interventions included intracystic drainage (49.0%), partial cystectomy (31.4%), and simple cystectomy (19.6%). The most frequent postoperative complications were bile leakage (6.9%) and anaphylaxis (4.9%). Recurrence occurred in 2.9%of patients, all of whom had undergone intracystic drainage. Crucially, the single reported mortality case was also associated with intracystic drainage rather than partial cystectomy. Younger children and female patients had significantly shorter hospital stays, although baseline cyst sizes were larger in females.

**Conclusion:**

Open surgery remains a safe and effective treatment for hepatic hydatid cysts in children, demonstrating a high recovery rate (99.0%) and low recurrence. However, intracystic drainage was associated with increased rates of bile leakage, recurrence, and mortality. Age and gender influenced hospital stay duration but not complication rates. Larger prospective studies are warranted to refine surgical strategies and improve long-term outcomes.

## Introduction

Cystic echinococcosis (CE), also known as hydatidosis, is a parasitic disease caused by *Echinococcus granulosus* and remains a major public health concern in many regions, particularly the Mediterranean, the Middle East, and Central Asia^[^1^]^. Carnivores like dogs act as definitive hosts, while livestock like sheep serve as intermediate hosts. Humans are accidental intermediate hosts; after ingestion of parasite eggs, the larvae penetrate the intestinal mucosa and typically migrate to the liver, followed by the lungs, where they develop into hydatid cysts^[^2^]^.

HIGHLIGHTS

- Open surgery achieves a 99% recovery rate for pediatric hepatic hydatid cysts.
- Intracystic drainage is associated with higher rates of bile leakage and recurrence.
- Female patients had larger cysts but shorter hospital stays.
- Age under 10 years was correlated with shorter hospitalization and lower vital sign values.
- Findings support the refinement of pediatric hydatid cyst surgical protocols.

Iran is recognized as an endemic area for CE, with provinces such as Khorasan, Hamedan, Markazi, and West and East Azerbaijan reported as high-prevalence regions^[^3^]^. Epidemiological studies show a prevalence of 5.8%in southern Iran, 2.2%in central regions, and up to 9%in northern and western provinces. The disease is more frequent in women and in rural populations, and accounts for approximately 1%of all surgical admissions in Iranian hospitals^[^3,4^]^.

Most hydatid cysts remain asymptomatic and are often detected incidentally during imaging for unrelated conditions. When symptoms do occur, they depend on the size, number, and location of the cysts, as well as the presence of complications^[^5^]^. If untreated, CE may result in serious outcomes, including peritoneal perforation (10–16%), secondary hydatidosis, cysto-biliary communications (9–25%), and even anaphylactic shock. In children, pulmonary involvement is relatively more common than in adults, although hepatic cysts remain a frequent presentation^[^6,7^]^. Spontaneous resolution, disappearance, or calcification of these cysts is rare, and medical or surgical intervention is usually required^[^8^]^.

Treatment strategies in children include open or laparoscopic surgery (radical or conservative), percutaneous aspiration–injection–reaspiration (PAIR), antiparasitic chemotherapy, and observation of inactive cysts^[^8^]^. Among these, open surgery remains widely practiced. However, recent studies have highlighted the potential advantages of minimally invasive approaches. For instance, Masood *et al* demonstrated that laparoscopic surgery can be an effective alternative to open surgery in pediatric hepatic hydatid disease, offering benefits such as a shorter hospital stay, earlier drain removal, less postoperative pain, and a quicker resumption of oral intake^[^9^]^.

Conversely, Khuroo *et al* emphasized the importance of a patient-centered, tailored approach and reaffirmed surgery as the most definitive intervention, with its ability to eradicate the parasite, prevent complications, and reduce the recurrence risk^[^10^]^. Within open surgical management, the debate between radical and conservative techniques persists. Sozuer *et al* concluded that no single method is universally optimal, stressing that the surgical strategy should be individualized according to cyst size, location, patient condition, and available resources^[^11^]^. Similarly, Vidoura *et al* suggested that conservative approaches are generally favored in endemic regions due to practicality and lower resource demands, whereas radical surgery is more appropriate in recurrent or complicated cases^[^12^]^.

Given the high prevalence of CE in Iran, the vulnerability of pediatric patients to complications and recurrence, and the lack of consistent evidence on optimal surgical approaches, this study was undertaken to evaluate the outcomes of open surgical treatment for hepatic hydatid cysts in children treated at university hospitals in Iran. This retrospective cohort study is reported in line with the STROCSS guidelines^[^13^]^.

## Materials and methods

### Study design and eligibility criteria

This retrospective cohort study was conducted with approval from the institutional ethics committee. Pediatric patients diagnosed with hepatic hydatid cysts and treated at two tertiary pediatric centers between 2017 and 2024 were eligible for inclusion. Medical records were retrospectively reviewed to identify children who underwent open surgical treatment during this period. Patients were included if they had hepatic cysts larger than 5cm with a non-multilocular morphology confirmed by ultrasonography (US). Exclusion criteria were cysts smaller than 5cm, multilocular cysts, or incomplete medical records.

### Data collection

Extracted data included demographic characteristics (age and sex), cyst features (size and location within the hepatic lobes), length of hospital stays, type and duration of surgery, preoperative liver function tests, and general clinical condition based on vital signs.

### Perioperative medical management

Patients scheduled for surgical management of hepatic hydatid cysts underwent standardized perioperative medical therapy. Preoperative antiparasitic treatment with oral albendazole was initiated at a dose of 10**–**15 mg/kg/day (divided into two equal doses, not exceeding 800mg/day) for a duration of 1**–**4 weeks prior to surgery to reduce cyst viability, sterilize the cyst contents, and minimize intraoperative spillage risk.

### Surgical techniques

**Figure 1.**
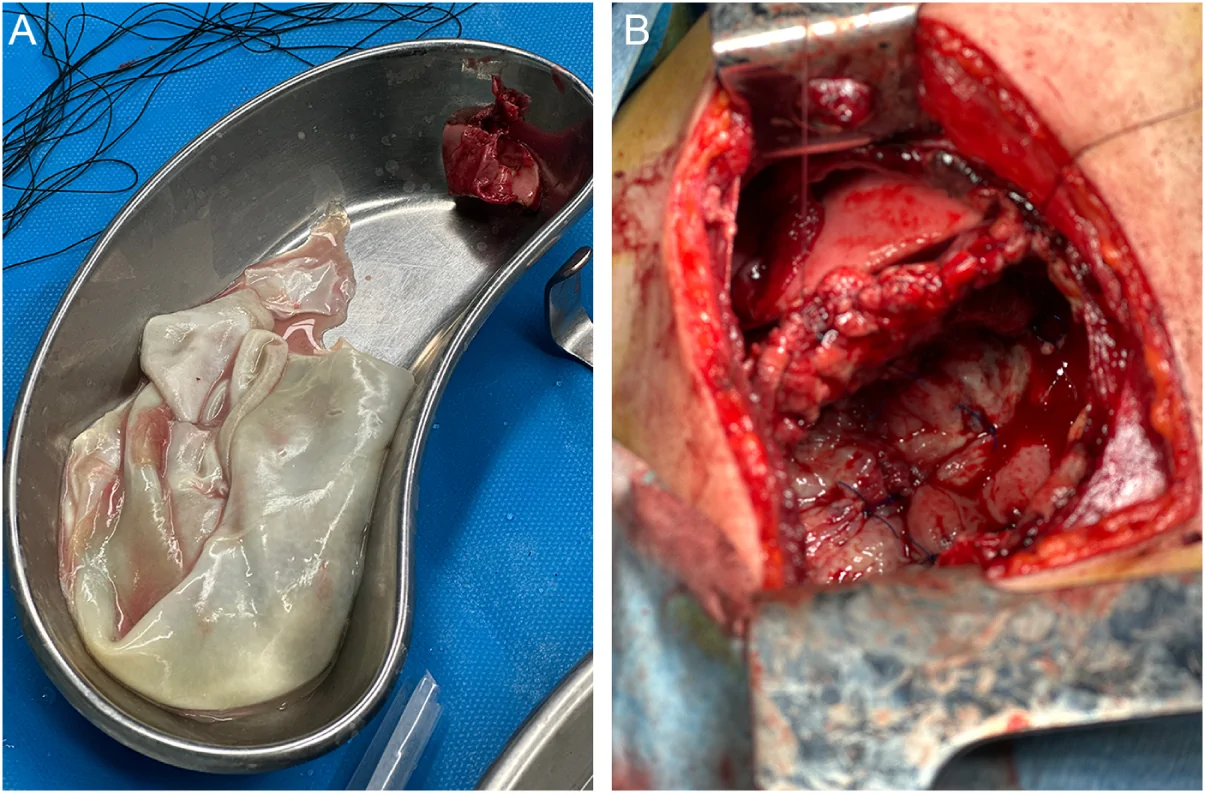
Intraoperative photographs of open surgery for a hepatic hydatid cyst. (A) Macroscopic appearance of the extracted endocyst (germinal layer) following removal. (B) Intraoperative view of the open surgical field demonstrating the residual hepatic cavity (pericyst) and subsequent cavity management.

The choice of surgical method was determined by cyst size, location, and the patient's condition. Figure 1 illustrates the intraoperative extraction of the endocyst and the management of the residual hepatic cavity during an open procedure. Techniques included:

- *Simple cystectomy*: complete cyst removal without hepatic tissue resection.
- *Partial cystectomy*: partial excision of the cyst wall combined with scolicidal irrigation.
- *Intracystic drainage*: aspiration and antiseptic irrigation, typically reserved for large or complex cysts.
- *Pericystectomy*: removal of the cyst along with surrounding liver tissue to reduce recurrence risk.

The selection of the surgical approach for each hepatic hydatid cyst was guided by a standardized clinical protocol evaluating several key factors. These primarily included the cyst’s size, anatomical location and proximity to vital structures, and the presence of complications, such as a biliary fistula, secondary infection, rupture, or compression of adjacent organs. In this study, all patients underwent open surgical management. This approach was chosen exclusively because it remains the safest and most effective standard of care for large or complicated cysts in children. Open surgery provides superior direct visualization and access through a standard abdominal incision, allowing the surgical team to completely evacuate the cyst contents and meticulously clear the area. Furthermore, it significantly reduces the risk of accidental spillage and intra-abdominal dissemination – a recognized limitation associated with the restricted working field of laparoscopic techniques. The decision between partial cystectomy and simple cystectomy was dictated by the same core criteria: the overall complexity and size of the cyst, along with the degree of pericystic inflammation and its adherence to surrounding functional liver tissue.

To ensure the absolute inactivation of any remaining parasite protoscoleces and to prevent intraoperative complications such as anaphylaxis or future recurrence, a scolicidal agent was routinely employed. The cyst cavity was sterilized using hypertonic saline (20%), which was injected and allowed adequate contact time prior to the complete aspiration and removal of the cyst contents.

### Outcome assessment

Early postoperative complications, including fever, bile leakage, surgical-site infections, pleural complications, and anaphylaxis, were systematically recorded. Recovery outcomes and mortality were also documented.

### Standardized definitions for postoperative complications

We used standard clinical criteria to consistently track and define postoperative complications. Based on the International Study Group of Liver Surgery (ISGLS) criteria, bile leakage was defined as a drain fluid bilirubin level at least 3times higher than the serum bilirubin on or after postoperative day 3. Surgical-site infections were identified using standard Centers for Disease Control and Prevention (CDC) guidelines. Postoperative fever was recorded if a patient’s core temperature reached ≥38.0 °C after the first 24hours following surgery. For patients showing respiratory symptoms, pleural complications (such as effusion or reactive atelectasis) were confirmed by chest X-ray. Finally, any severe complications requiring critical care were classified according to standard ICU admission criteria.

### Follow-up protocol

Follow-up data were collected retrospectively by reviewing patient electronic medical records, outpatient clinical notes, and radiological archives. All patients included in the final analysis were monitored for a minimum period of 12months, with a total follow-up range of 12–24 months. The standard follow-up schedule documented in the patient records consisted of regular clinical examinations and abdominal US to screen for recurrence or late-onset complications. These assessments were typically performed at 3, 6, and 12months post-discharge, and then annually thereafter. Remote follow-up was utilized in cases where in-person visits were not possible to assess symptom status and quality of life. A retrospective review of electronic medical records and imaging databases was also conducted to ensure comprehensive capture of any secondary interventions or readmissions occurring throughout the study period.

### Intensive care unit admission criteria

Patients were considered for admission to the intensive care unit (ICU) based on the presence of specific complications or significant physiological derangement. Indications for ICU admission included:

- *Hemodynamic instability*: Persistent hypotension (systolic blood pressure <90 mmHg) despite adequate fluid resuscitation and vasopressor support, or the need for high-dose vasopressor therapy.
- *Respiratory compromise*: Mechanical ventilation requirement, significant hypoxemia (PaO_2_/FiO_2_ ratio <200 mmHg), or severe metabolic acidosis with respiratory compensation.

- *Severe sepsis or septic shock*: Identified by an increase in the Sequential Organ Failure Assessment (SOFA) score and Systemic Inflammatory Response Syndrome (SIRS), necessitating advanced hemodynamic support.
- *Postoperative bleeding*: Significant ongoing blood loss requiring massive transfusion or reoperation.
- *Electrolyte and metabolic disturbances*: Severe electrolyte imbalances or metabolic derangements (e.g., severe hyperglycemia or hypoglycemia) requiring initial management.

### Imaging modalities

**Figure 2.**
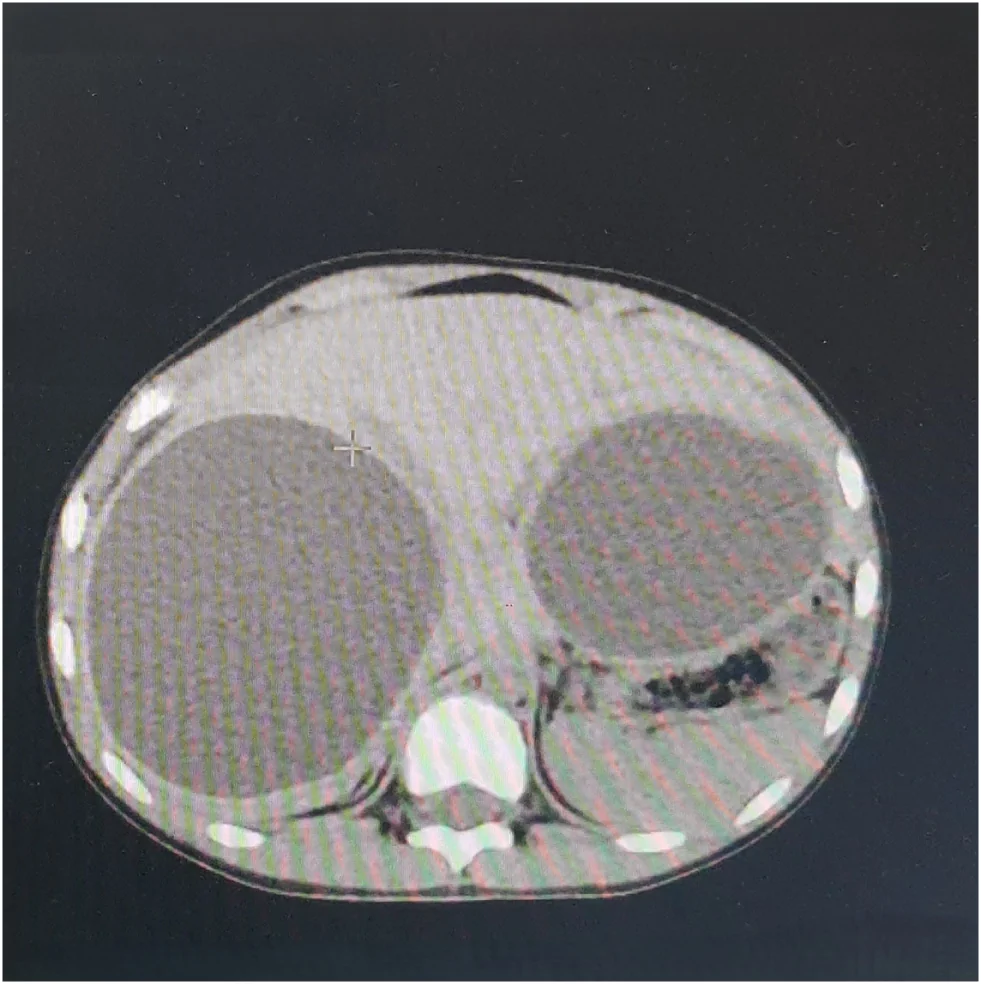
Axial computed tomography (CT) scan of the abdomen demonstrating multiple large, well-defined, unilocular cystic lesions in the hepatic parenchyma, characteristic of giant hepatic hydatid cysts. The cysts exhibit homogeneous fluid density without internal septations, causing significant displacement of surrounding structures.

In this study, US was used as the initial imaging modality to confirm the presence of the lesion. The size, location, and relationship with adjacent structures were evaluated with this modality. When additional anatomical details were required for further evaluation and surgical planning, computed tomography (CT) was performed (Fig. 2). The extend of the lesion, involvement of surrounding tissues, and potential operative challenges were assessed using CT. During the postoperative follow-up, US was used to assess for recurrence, residual lesions, or possible complications. The choice of imaging modality was based on patient-specific and clinical considerations.

### Statistical methods and sample size

Based on the study objectives and applying Cochran’s formula, the required sample size was calculated using a prevalence of open surgical cases reported in prior studies (*P* = 0.15) at a 95% confidence level. This yielded an estimated sample size of 100participants. To accommodate a potential dropout rate of <10%, the final sample size was set at N=102 patients.

Data were analyzed using SPSS software (version 24). The Kolmogorov–Smirnov test was used to assess the normality of continuous variables. Quantitative data were presented as mean ±standard deviation, while categorical variables were summarized as frequencies and percentages. Associations between categorical variables were evaluated using the Chi-square test or Fisher’s exact test, depending on expected cell frequencies. A two-tailed *P*-value of <0.05 was considered statistically significant.

### Ethical considerations

All ethical principles were strictly observed in this study. Data collected from patient records were kept confidential and used exclusively for research purposes. To ensure privacy, all identifying information was removed, and the data were anonymized and recorded in coded form.

## Results

A total of 102 pediatric patients with hepatic hydatid cysts who underwent open surgery were included in the analysis. Of these, 64patients (62.7%) were male, and 38patients (37.3%) were female.

The mean age at surgery was 9.08±4.80years (range: 1**–**18 years). Most patients (n=68, 66.7%) were younger than 10years, while 34patients (33.3%) were aged years or older. With respect to hepatic lobe involvement, the right lobe was affected in 68 cases (66.7%), the left lobe in 17cases (16.7%), and both lobes in 17cases (16.7%).

The mean cyst size was 7.13±2.98cm (range: 1**–**15 cm). The mean length of hospital stay for the retrospective cohort was 6.33±5.37days, ranging from 1to 40 days. Prolonged hospitalizations were primarily due to the management of postoperative complications, such as biliary fistulas or secondary infections, particularly in patients characterized by older age (≥10 years) and male sex, who statistically demonstrated significantly longer recovery periods in our study. The mean operative time was 119.21±36.36minutes (range: 55**–**230 minutes).

Regarding surgical techniques, 50patients (49.0%) underwent intracystic drainage alone, 32patients (31.4%) received a combined procedure involving drainage with partial cystectomy, and 20patients (19.6%) underwent simple cystectomy.

Preoperative liver function tests revealed mean serum alkaline phosphatase (ALP) levels of 283.23±374.52IU/L (range: 0**–**2610 IU/L), alanine aminotransferase (ALT) of 54.39±136.49IU/L (range: 0**–**870 IU/L), and aspartate aminotransferase (AST) of 80.35±295.73IU/L (range: 0**–**2105 IU/L). Mean direct bilirubin was 0.10±0.28mg/dL (range: 0**–**1.8 mg/dL), while mean total bilirubin was 0.26±0.49mg/dL (range: 0**–**3 mg/dL). Vital signs at admission demonstrated a mean systolic blood pressure of 104.35±9.52mmHg (range: 60**–**121 mmHg), mean diastolic blood pressure of 68.58±5.63mmHg (range: 40**–**78 mmHg), and a mean heart rate of 92.62±7.92beats per minute (range: 78**–**130 bpm) (Table 1).

**Table 1:** Clinical and demographic characteristics of pediatric patients with hepatic hydatid cysts.

Variable	Mean ± SD or *n* (%)
Demographic features	
Age < 10 years	6866.7%
Age ≥10 years	3433.3%
Surgical features	
Involved hepatic lobe – right	6866.7%
Involved hepatic lobe – left	1716.7%
Involved hepatic lobe – both lobes	1716.7%
Clinical and procedural characteristics	
Cyst size (cm)	7.13±2.98
Duration of surgery (minutes)	119.2±36.36
Length of hospital stay (days)	6.33±5.37
Type of surgery	
Intracystic drainage	5049.0%
Partial cystectomy	3231.4%
Simple cystectomy	2019.6%
Liver function tests (pre-op)	
ALP (IU/L)	283.23±374.52
ALT (IU/L)	54.39±136.49
AST (IU/L)	80.35±295.73
Direct bilirubin (mg/dL)	0.10±0.28
Total bilirubin (mg/dL)	0.26±0.49
Vital signs	
Systolic blood pressure (mmHg)	104.4±9.5
Diastolic blood pressure (mmHg)	68.6±5.6
Heart rate (bpm)	92.6±7.9

### Postoperative complications and outcomes

Early postoperative complications included diaphragmatic injury in 2 patients (2.0%), bile leakage in 7patients (6.9%), and anaphylaxis in 5patients (4.9%). Hypotension, postoperative pain, and other nonspecific symptoms were each reported in 1patient (1.0%).

In terms of long-term outcomes, recurrence of hepatic hydatid cysts was documented in 3patients (2.9%). A detailed review of these cases revealed the following:

- *Case 1:* Recurrence occurred at 14months post-surgery. The initial cyst was located in the right lobe, Segment V, and the recurrence was identified in the same lobe.
- *Case 2:* Recurrence occurred at 8months post-surgery. The initial cyst was located in the right lobe, Segment VI, and the recurrence was identified in Segments VI and VII.
- *Case 3:* Recurrence occurred at 6months post-surgery. The initial cyst was located in the right lobe, Segments V and VIII, and the recurrence was identified in the same location.

Among the 3 observed recurrences, two patients had strictly adhered to the prescribed postoperative albendazole therapy (administered at 10**–**15 mg/kg/day for 1**–**3 months).

Unfortunately, 1 patient (1.0%) died following surgery. The mortality was secondary to a severe infection of the recurrent cyst and the initial surgical cavity, which ruptured into the biliary tree. This complication led to acute suppurative cholangitis, overwhelming sepsis, and ultimately multi-organ failure, despite aggressive resuscitative efforts and medical management (Table 2).

**Table 2:** Postoperative complications and outcomes in pediatric patients with hepatic hydatid cysts.

Variable	Number (*n*)	Percentage (%)
Complications		
Diaphragmatic injury	2	2.0
Bile leakage	7	6.9
Anaphylaxis	5	4.9
Hypotension	1	1.0
Pain	1	1.0
Other symptoms	1	1.0
Outcomes		
Recurrence	3	2.9
Mortality	1	1.0

### Age-stratified outcomes

Analysis of the relationship between age and clinical variables revealed no statistically significant differences in mean cyst size or surgical duration between children younger than 10years and those aged 10years or older (pgt0.05). However, the mean length of hospital stay was significantly shorter in the younger age group (plt;0.05). Preoperative liver function tests – including ALP, ALT, AST, direct bilirubin, and total bilirubin – showed no significant differences between the two groups (pgt0.05). In contrast, vital signs demonstrated age-related variation: systolic and diastolic blood pressure, as well as heart rate, were significantly lower in patients younger than 10years compared with older children (plt0.05). No statistically significant associations were identified between age group and hepatic lobe involvement, type of surgery, postoperative complications, recurrence, or mortality (pgt0.05) (Table 3).

**Table 3:** Age-stratified clinical, surgical, and postoperative outcomes in pediatric hepatic hydatid disease.

Variable	<10 Years (*n* = 68) (Mean ± SD / *n* (%))	≥10 Years (*n* = 34) (Mean ± SD / *n* (%))	*P*-value
Cyst size (cm)	7.35±2.81	6.50±3.25	0.323
Length of hospital stay (days)	5.13±2.34	8.73±8.25	0.014∗
Surgery duration (min)	117.05±2.34	123.52±34.27	0.432
Liver function (preoperative)			
ALP (U/L)	393.21±258.61	334.13±332.47	0.178
ALT (U/L)	156.64±57.45	84.54±48.26	0.383
AST (U/L)	358.03±102.98	65.25±35.08	0.409
Direct bilirubin (mg/dL)	0.21±0.08	0.39±0.15	0.772
Total bilirubin (mg/dL)	0.36±0.21	0.68±0.36	0.600
Vital signs			
Systolic blood pressure (mmHg)	100.16±7.82	112.73±6.71	0.001∗
Diastolic blood pressure (mmHg)	67.02±5.59	71.70±4.30	0.001∗
Heart rate (bpm)	95.17±7.57	87.52±5.96	0.001∗
Involved hepatic lobe			0.085
Right	41(60.3%)	27(79.4%)	
Left	12(17.6%)	5(14.7%)	
Both lobes	15(22.1%)	2(5.9%)	
Type of surgery			0.750
Intracystic drainage	32(47.1%)	18(52.9%)	
Partial cystectomy	23(33.8%)	9(26.5%)	
Simple cystectomy	13(19.1%)	7(20.6%)	
Postoperative complications			
Diaphragmatic injury	2(2.9%)	0(0.0%)	0.313
Bile leakage	4(5.9%)	3(8.8%)	0.580
Anaphylaxis	3(4.4%)	2(5.9%)	0.746
Hypotension	1(1.5%)	0(0.0%)	0.477
Pain	1(1.5%)	0(0.0%)	0.477
Other symptoms	1(1.5%)	0(0.0%)	0.477
Recurrence	1(1.5%)	2(5.9%)n=64	0.214
Mortality	1(1.5%)	0(0.0%)	0.477

### Gender-based outcomes

The mean hospital stay was significantly shorter in female patients compared with males (plt;0.05), although the mean cyst size was significantly larger in females (plt;0.05). No statistically significant sex-related differences were observed for surgical duration, preoperative liver function parameters, or vital signs (pgt0.05). Furthermore, gender was not significantly associated with hepatic lobe involvement, type of surgery, postoperative complications, recurrence, or mortality (pgt0.05) (Table 4).

**Table 4:** Gender-based clinical, surgical, and postoperative outcomes in pediatric hepatic hydatid disease.

Variable	Male (*n* = 64) (Mean ± SD / *n* (%))	Female (*n* = 38) (Mean ± SD / *n* (%))	*P*-value
Cyst size (cm)	6.23±2.40	9.08±3.28	0.003∗
Length of hospital stay (days)	6.96±6.23	5.26±3.25	0.014∗
Surgery duration (min)	119.68±37.37	118.42±35.06	0.975
Liver function (preoperative)			
ALP (U/L)	294.35±423.34	264.50±277.78	0.775
ALT (U/L)	47.39±126.17	66.18±153.37	0.607
AST (U/L)	70.00±262.48	97.78±347.75	0.829
Direct bilirubin (mg/dL)	0.11±0.30	0.10±0.26	0.743
Total bilirubin (mg/dL)	0.27±0.47	0.24±0.54	0.601
Vital signs			
Systolic blood pressure (mmHg)	104.07±10.59	104.81±7.50	0.800
Diastolic blood pressure (mmHg)	68.25±6.18	69.15±4.57	0.593
Heart rate (bpm)	93.45±8.93	91.23±5.69	0.505
Involved hepatic lobe			0.097
Right	47(73.4%)	21(55.3%)	
Left	7(10.9%)	10(26.3%)	
Both lobes	10(15.6%)	7(18.4%)	
Type of surgery			0.387
Intracystic drainage	34(53.1%)	16(42.1%)	
Partial cystectomy	17(26.6%)	15(39.5%)	
Simple cystectomy	13(20.3%)	7(18.4%)	
Postoperative complications			
Diaphragmatic injury	1(1.6%)	1(2.6%)	0.707
Bile leakage	6(9.4%)	1(2.6%)	0.193
Anaphylaxis	4(6.3%)	1(2.6%)	0.413
Hypotension	1(1.6%)	0(0.0%)	0.439
Pain	1(1.6%)	0(0.0%)	0.439
Other symptoms	1(1.6%)	0(0.0%)	0.439
Recurrence	2(3.1%)	1(2.6%)	0.887
Mortality	1(1.6%)	0(0.0%)	0.439

### Surgical technique and outcomes

The type of surgical procedure was not significantly associated with overall postoperative complications, recurrence, or mortality (pgt0.05). However, all 3cases of recurrence were observed in patients treated with intracystic drainage. Similarly, the single mortality occurred following intracystic drainage. Additionally, among the 7cases of bile leakage, 5(10.0% of the drainage cohort) were linked to intracystic drainage (Table 5).

**Table 5:** Association between surgical techniques and postoperative outcomes in pediatric hepatic hydatid cyst surgery.

Variable	Intracystic drainage (*n* = 50) *n* (%)	Partial cystectomy (*n* = 32) *n* (%)	Simple cystectomy (*n* = 20) *n* (%)	*P*-Value
Diaphragmatic injury	0(0.0%)	1(3.1%)	0(0.0%)	0.335
Bile Leakage	5(10.0%)	1(3.1%)	1(5.0%)	0.454
Anaphylaxis	1(2.0%)	1(3.1%)	3(15.0%)	0.064
Hypotension	0(0.0%)	1(3.1%)	0(0.0%)	0.331
Pain	0(0.0%)	1(3.1%)	0(0.0%)	0.331
Recurrence	3(6.0%)	0(0.0%)	0(0.0%)	0.200
Mortality	1(2.0%)	0(0.0%)	0(0.0%)	0.331

## Discussion

In this study, 102pediatric patients with hepatic hydatid cysts treated via open surgery were evaluated. Most cases (66.7%) involved the right hepatic lobe. Surgical procedures included intracystic drainage (49.0%), partial cystectomy (31.4%), and simple cystectomy (19.6%). The most common early complications were biliary leakage (6.9%) and anaphylaxis (4.9%). Recurrences occurred in 2.9%of patients, while one mortality was reported, corresponding to a 99%recovery rate. Younger children had significantly shorter hospital stays compared to older children, and female patients experienced shorter hospital stays despite having larger cysts. No statistically significant association was found between surgical technique and complication rates, recurrence, or mortality; however, all recurrences were linked to intracystic drainage, and the single mortality was observed after intracystic drainage. Among 7instances of biliary leakage, 5occurred following internal drainage interventions.

The choice between surgical techniques in our retrospective cohort including partial cystectomy (31.4%) and simple cystectomy (19.6%) was guided by the cyst’s characteristics, anatomical location, and intraoperative findings. Partial cystectomy was favored for larger, deeply situated cysts, or those near major vascular and biliary structures where complete excision posed a high risk of major hemorrhage or ductal injury. Conversely, simple cystectomy was typically performed for smaller, peripherally located cysts, or those with well-defined, uninflamed planes, allowing for safe and complete enucleation without compromising adjacent vital structures.

Hydatid disease continues to be a significant pediatric health problem in endemic regions, with the liver being the most frequently affected organ^[^14^]^. Surgery remains the mainstay of treatment, and cystectomy is the most commonly performed procedure due to its favorable outcomes and relatively low complication rates^[^15^]^. Radical approaches, such as complete cystectomy with pericystectomy, may reduce recurrence, but conservative strategies – such as partial cystectomy with drainage, omentoplasty, or marsupialization – are often favored because they carry fewer immediate risks^[^16^]^. The disease often remains asymptomatic for years, but once diagnosed, treatment is critical to prevent life-threatening complications such as rupture, secondary hydatidosis, and cysto-biliary communications^[^17^]^. Diagnosis generally relies on abdominal US, while newer imaging modalities have improved sensitivity and diagnostic accuracy^[^18^]^.

The complication profile of hydatid cysts has been well described, with biliary rupture and recurrence among the most common issues^[^19^]^. In our retrospective cohort, biliary leakage (6.9%) and anaphylaxis (4.9%) were the leading postoperative complications, consistent with reported rates of 6%**–**17%. Although no statistically significant link was observed between surgical technique and adverse outcomes, recurrence occurred exclusively in patients undergoing intracystic drainage, and mortality was associated with intracystic drainage. These findings reinforce that drainage procedures may carry higher risks of bile leakage and recurrence.

Rupture of hydatid cysts into the peritoneal cavity, although rare, represents a severe complication with acute and long-term consequences, including peritoneal hydatidosis. Anaphylaxis is another recognized risk, particularly with PAIR or surgical manipulation, caused by cyst fluid entering the systemic circulation. Interestingly, one study reported no cases of anaphylaxis in their series of over 200children, highlighting how differences in sample size and surgeon expertise may influence outcomes^[^14–16^]^.

Open surgery continues to dominate the management of abdominal hydatidosis, as laparoscopic techniques remain less frequently used. Studies by Gollackner *et al* and Cirenei *et al* demonstrated that open procedures are associated with lower rates of recurrence and mortality^[^17,18^]^. Complete cystectomy remains the preferred approach when technically feasible, although cysts in the right hepatic lobe can sometimes be removed only with difficulty, increasing the risk of postoperative infection or recurrence. Cysts in the right hepatic lobe are typically excised without compression, rendering the residual parenchyma more susceptible to infection and subsequent cyst recurrence, thereby reinforcing the notion of limited efficacy of total cystectomy^[^18,19^]^. Razumovsky *et al* reported favorable outcomes with total cystectomy, whereas partial cystectomy may still be useful in preserving liver parenchyma and reducing intraoperative risk^[^20,21^]^. Quality-of-life studies, such as those by De Reuver *et al*, suggest that partial cystectomy may provide symptom relief, although its long-term durability remains uncertain^[^22^]^. Jarvis *et al*^[^23^]^ demonstrated that cyst excision with adjunctive omentoplasty effectively alleviates pressure- and inflammation-related symptoms. Further investigations are warranted to elucidate the outcomes of partial cystectomy in relation to quality-of-life metrics and inflammatory parameters**^[^**23,24^]^.

Our results are consistent with previous reports indicating that while radical surgery reduces recurrence, conservative procedures are often more practical in pediatric populations. Similarly, Motie *et al* and Gomez *et al* advocated for open surgery due to its balance of effectiveness and safety^[^25,26^]^. Tagliacozzo *et al*^[^27^]^ concluded that radical resection should be confined to exceptional circumstances to prevent the unwarranted removal of intact hepatic parenchyma. Other large-scale studies, including those by Georgiou *et al* and Secchi *et al*, confirm that open surgery achieves superior outcomes compared to conservative management^[^18,28^]^.

Open surgical intervention provides the most definitive management by ensuring complete cyst excision and eliminating the risk of recurrence^[^29^]^. Whenever feasible, total pericystectomy is favored, as it minimizes injury to healthy hepatic parenchyma; however, anatomical resection may be required in cases of extensive cystic involvement. Given the benign nature of hydatid disease, the preservation of functional liver tissue should remain a priority^[^30^]^.

When comparing surgical techniques (Table 5), we found no statistically significant differences regarding postoperative complications, recurrence, or mortality (pgt0.05). While our data do not suggest the definitive superiority of one approach, it is noteworthy that all recorded recurrences and most bile leakage cases occurred in patients who underwent intracystic drainage. These findings suggest that while all three techniques are viable options in the surgical management of pediatric hepatic hydatid cysts, the choice of procedure should be individualized based on the cyst’s anatomical characteristics and the surgeon’s expertise, particularly when weighing the risks of biliary complications and long-term recurrence.

In summary, the open surgical management of hepatic hydatid cysts carries risks such as biliary leakage and anaphylactic reactions. Although complete cystectomy is considered the standard approach, accumulating evidence indicates that partial cystectomy may offer comparable efficacy with fewer hepatobiliary complications, particularly in pediatric patients. Treatment strategies should account for patient age, general health, cyst characteristics, and surgical expertise^[^24^]^.

In the pediatric population, open procedures typically involve either total or partial cystectomy. Younger children tend to exhibit shorter hospitalization and faster recovery compared to older cohorts^[^31^]^. In our study, patients under 10years of age had significantly reduced hospital stays and lower vital parameters than those aged ≥10years. The observed shorter hospital stays in female patients, despite their having larger cyst sizes, may reflect differences in postoperative recovery patterns rather than disease severity. Previous studies have suggested that female patients often demonstrate better adherence to postoperative care recommendations and faster recovery after abdominal surgery, which might contribute to shorter hospitalization. Moreover, social and behavioral factors, such as earlier presentation to medical care or a lower threshold for symptom reporting among women, may partly explain this finding^[^32^]^.

Epidemiological data suggest that hepatic hydatid cysts are more prevalent among males, likely due to greater occupational exposure^[^33,34^]^. Although less frequently affected, female patients may experience differential outcomes influenced by hormonal and physiologic factors^[^35^]^. Consistent with this, our findings indicated that cysts in girls were significantly larger but were associated with shorter hospitalization. Age and sex thus appear to modulate postoperative recovery and complication rates.

Overall, our results contribute valuable data on open surgical outcomes for pediatric hepatic hydatid cysts in an endemic region. By identifying complication patterns and highlighting procedure-specific risks, this study supports the refinement of surgical strategies. The long-term evaluation of liver function, growth, and quality of life is essential for a comprehensive assessment of outcomes. These insights may guide future treatment protocols and help optimize surgical decision-making for pediatric patients.

### Limitations and strengths

This study is the first from Mashhad to evaluate the outcomes of open surgical techniques for pediatric hepatic hydatid cysts, providing valuable data from an endemic region. However, several limitations should be noted. The relatively small sample size and limited follow-up duration may have reduced the ability to detect late recurrences or long-term complications. In addition, variations in surgical techniques and in the reporting of complications present challenges to standardization and to comparisons across cohort studies.

Another limitation of this study is the lack of direct comparison between open surgical techniques and minimally invasive approaches like PAIR. While some patients met the morphological criteria for PAIR, the surgical team opted for open surgery to ensure complete management of the cyst wall and reduce the risk of recurrence. Future prospective studies are needed to compare the long-term efficacy of PAIR with that of open surgery in pediatric populations with similar cyst characteristics.

Although the laparoscopic management of hepatic hydatid cysts has been reported with favorable outcomes in some centers, our study focuses on the results of open surgical intervention. The selection of open surgery in this retrospective cohort was largely dictated by the size of the cysts, their anatomical location, and an institutional preference to ensure comprehensive exploration and management of cysts in pediatric patients. Future research comparing laparoscopic and open approaches, specifically within our pediatric centers, would be valuable to further refine surgical indications.

Despite these limitations, the study’s strengths include its relatively large pediatric retrospective cohort for a rare condition, systematic evaluation of multiple surgical approaches, and detailed analysis of postoperative outcomes. Together, these findings contribute to the growing body of evidence on hydatid disease in children and highlight the need for larger, multicenter, and methodologically robust studies to confirm and extend these results.

## Conclusion

This study evaluated 102pediatric patients with hepatic hydatid cysts treated via open surgery, with the right hepatic lobe being the most affected site. Surgical approaches included intracystic drainage, partial cystectomy, and simple cystectomy. The most frequent complications were bile leakage (6.9%) and anaphylaxis (4.9%), while recurrence occurred in 2.9%of cases and one patient (1.0%) died, yielding an overall recovery rate of 99%. Younger children and female patients experienced shorter hospital stays, although cyst size was significantly larger in females.

While surgical technique was not significantly associated with complication rates, intracystic drainage was associated with higher rates of bile leakage and recurrence, and the single mortality occurred following intracystic drainage. Based on our findings, open surgery, encompassing both partial and simple cystectomy, presents a robust and effective treatment strategy for pediatric hepatic hydatid cysts. This approach provides a high overall recovery rate and a manageable complication profile, particularly for complex cases or in settings where less invasive techniques may not be feasible or optimal. While acknowledging the benefits of PAIR and laparoscopic approaches described in the literature, our experience highlights the continued value and reliability of open surgery. Therefore, individualized surgical planning and the refinement of operative strategies remain essential for improving outcomes. Larger multicenter studies are warranted to further establish standardized protocols and optimize treatment.

## Data Availability

The data sets used and analyzed during the present study are available from the corresponding author on reasonable request.

## Research registration unique identifying number (UIN)

This study is retrospective in design and therefore registration in a public research registry was not required.
